# High frequency of diastolic dysfunction in a population-based cohort of elderly women - but poor association with the symptom dyspnea

**DOI:** 10.1186/1471-2318-11-71

**Published:** 2011-11-02

**Authors:** Alfried Germing, Michael Gotzmann, Tamara Schikowski, Andrea Vierkötter, Ulrich Ranft, Ursula Krämer, Andreas Mügge

**Affiliations:** 1 Medizinische Klinik II (Kardiologie & Angiologie), Berufsgenossenschaftliches Universitätsklinikum Bergmannsheil, Ruhr-Universität Bochum, Bochum, Germany; 2 Institut für Umweltmedizinische Forschung (IUF) an der Heinrich Heine-Universität, Düsseldorf, Germany

## Abstract

**Background:**

The European Society of Cardiology recently proposed a new algorithm "How to diagnose heart failure with normal ejection fraction". Central element of the diagnostic strategy is the demonstration of diastolic dysfunction, either by tissue Doppler-derived indices in first line, or in second line by a combination of elevated blood levels of natriuretic peptide with abnormal tissue Doppler findings. We thought to use this diagnostic flowchart in a population-based cohort of elderly women, in whom the prevalence of diastolic dysfunction and heart failure is believed to be high. The purpose was to evaluate the association of dyspnea with the presence of diastolic dysfunction.

**Methods:**

The study cohort recruited from a cross-sectional follow-up examination of the SALIA cohort (study on the influence of air pollution on lung function, inflammation, and aging). Participants with cardiac or pulmonary disease were excluded, 291 participants formed the final study group (all women, age range 69 to 79 years, all in sinus rhythm, LV ejection fraction > 50%, LV enddiastolic volume index < 97 mL/m^2^). Quality of life was assessed by the Minnesota living with heart failure questionnaire, and actual symptoms by a structural questionnaire; the examination consisted of a physical examination, measurement of B-type natriuretic peptide, ECG and tissue Doppler echocardiography. Diastolic dysfunction was assumed when the E/E' ratio exceeded 15 as derived from tissue Doppler. In case, tissue Doppler yielded an E/E' ratio ranging from 8 to 15, additional non-invasive parameters had to be fulfilled: left atrial volume index > 40 ml/m^2 ^body surface, or left ventricular mass index > 122 g/m^2 ^body surface, or transmitral E/A ratio < 0.5 plus deceleration time > 280 ms, or blood level of brain natriuretic peptide (BNP) > 200 pg/mL.

**Results:**

The examinations were concordant with the presence of diastolic dysfunction in 122/291 participants (41.9%). The diagnosis based in 94% of cases on two criteria: in 50 cases on the criterion "E/E' ratio > 15", and in 65 cases on the criterion "15 > E/E'>8 and LV mass index > 122 g/m^2^". The participants with diastolic dysfunction had on average a higher body mass index, more frequent a history of arterial hypertension and of hospitalization for congestive heart failure, poorer quality of life, and higher BNP blood levels as compared to those participants without signs of diastolic dysfunction. The number of participants complaining exertional dyspnea, however, was similar distributed among the subgroups with and without signs of diastolic dysfunction (40.2 vs 40.8%; p = n.s). In a logistic regression model, the symptom dyspnea was best predicted by systolic pulmonary artery pressure, followed by left atrial volume index, BNP, and body mass index.

**Conclusion:**

The demonstration of diastolic dysfunction showed only a poor association with the symptom dyspnea in a cohort of elderly women with otherwise normal systolic function. Additional structural or hemodynamic changes are necessary to "explain" the symptom dyspnea. It is unclear whether these additional factors are secondary to a more advanced stage of diastolic dysfunction, or are related to cardiovascular co-morbidities, or both.

## Background

In the last two decades, there is growing evidence of prevalence and prognosis of heart failure with normal ejection fraction (HFNEF), also referred to as diastolic heart failure [1]. HFNEF is characterized by symptoms of heart failure, signs of elevated ventricular filling pressures and impaired relaxation in presence of normal systolic function [2]. In 2007, the Heart Failure and Echocardiography Associations of the European Society of Cardiology proposed in a consensus statement a diagnostic flowchart providing a strategy on "How to diagnose HFNEF" [3]. The diagnostic strategy is intended for patients suspected of having HFNEF and is based on the positive predictive value of successive examinations. According to this consensus statement, the diagnosis of HFNEF requires a.) symptoms or signs of heart failure, b.) normal or mildly abnormal systolic LV function, and c.) evidence of diastolic dysfunction.

Diagnostic evidence of diastolic dysfunction can be obtained invasively, or - which is probably more accepted in clinical routine - non-invasively by tissue Doppler. A central parameter derived from tissue Doppler is the ratio E/E' (early diastolic peak filling velocity as derived from transmitral Doppler/early diastolic peak velocity of the mitral annulus as derived from tissue Doppler). When this ratio exceeds 15, the LV filling pressures are elevated, and - in the presence of normal systolic LV function - highly suggestive for the presence of diastolic dysfunction [4,5]. A "grey zone" is the E/E' ratio ranging from 8 to 15 suggestive but nondiagnostic for the presence of diastolic dysfunction. In this case, the consensus statement suggests implementing additional non-invasive investigations, such as left atrial volume measurement, left ventricular mass measurements, combined assessment of transmitral E/A ratio and deceleration time, or the implementation of blood levels of B-type natriuretic peptides [3].

We thought to use this diagnostic flowchart in a population-based cohort of elderly women, in whom the prevalence of diastolic dysfunction and heart failure is believed to be high [6,7]. The purpose was to evaluate the association of dyspnea with the presence of diastolic dysfunction.

## Methods

### SALIA cohort population

The SALIA cohort (Study on the influence of Air population on Lung function, inflammation and Aging) was initiated as part of the Environmental Health Surveys introduced by the North Rhine Westfalia government between 1985 and 1994. The study population comprised 4874 women aged 55 at the time of entering the study who were living in predefined residential areas. The study areas were chosen from the highly industrialized Ruhr region which represents a range of high-polluted areas and two rural counties in the north-west of the Ruhr region as reference areas [8,9]. In a follow-up of SALIA cohort from April 2007 to November 2008, 708 women from the Ruhr region cities Duisburg, Dortmund, Essen and Gelsenkirchen and the rural county Borken who survived and agreed in 2006 to participate in a follow-up were invited in a randomised manner and, at least 402 women participated. The protocol of the study was approved by the Medical Ethic Committee of the Ruhr University Bochum. All participants gave informed written consent.

### Cardiovascular study cohort

A cross-sectional cardiovascular examination was realized in 344 of 402 participants (86%). The examination consisted of medical history, physical examination, measurement of B-type natriuretic peptide, electrocardiography, and echocardiography.

Exclusion criteria for further analysis were a.) previous heart surgery and/or PCI (n = 5), b.) severe heart valve disease (n = 6), c.) typical angina pectoris ≥ II CCS (n = 3), d.) previous or actual atrial fibrillation or flutter (n = 8), e.) permanent pacemaker stimulation (n = 3), and f.) poor image quality in the echocardiographic examination (n = 10). Nine participants were excluded with a LV EF ≤ 50% and/or LVEDVI ≥ 97 mL/m^2^; further 9 participants were excluded with pulmonary diseases requiring a specific therapy. These latter exclusion criteria were chosen to focus on participants with a normal left ventricular systolic function and normal left ventricular enddiastolic diameter, and to exclude those participants with a significant pulmonary disease. Thus, a total of 291 participants formed the final study group.

### Medical history, quality of life and physical examination

In a structured interview each participant was asked for detailed medical history, particularly for cardiovascular risk factors, cardiovascular diseases and medication. The Minnesota living with heart failure questionnaire (MLHFQ) was used to assess quality of life [10]. Summation of the responses yielded the total MLHFQ score for each patient. The test ranges between 0 and 110, whereas higher score indicates a poorer quality of life.

Dyspnea was assessed by combining results of the standardized questionnaire and an interview. Participants were asked to asses their degree of functional impairment due to dyspnea and level of activity. With this, dyspnea was categorized according to the New York Heart Association classification.

Physical examination comprised measurements of blood pressure, height, and weight. Arterial hypertension was diagnosed by medical history, medication and blood pressure measurement (systolic pressure > 140 mmHg and/or diastolic blood pressure > 90 mmHg).

### Measurement of B-type natriuretic peptide and electrocardiography

Plasma B-type (brain) natriuretic peptide levels (BNP) were measured at the same day as electrocardiography and echocardiography. The blood samples were collected in EDTA-containing tubes. After prompt centrifugation, BNP was measured using a chemiluminescent immunoassay kit (Biosite Triage, San Diego, CA, USA). Electrocardiography was analyzed for arrhythmias.

### Echocardiography

Transthoracic echocardiography was performed according to the guidelines of the American Society of Echocardiography [11] using a digital ultrasound scanner (Vivid 7, General Electrics, Horton, Norway). Data from three cardiac cycles were analyzed. An experienced cardiologist, blinded to the participant's clinical data, performed the ultrasound examination and interpreted the results. Left ventricular diameter, posterior wall and septal thickness, aortic root and left atrial diameter were measured by M-Mode from the parasternal views. Left atrial volume was measured by manual tracing of end-systolic endocardial borders using the apical 4-chamber view. Left ventricular myocardial mass was calculated according to the Devereux formula. The Quinones formula was used for measurement of the left ventricular ejection fraction. Values were averaged for each patient. Right atrial and ventricular dimensions were measured in apical 4-chamber and parasternal short-axis views, tricuspid annular plane systolic excursion (TAPSE) was measured by M-mode recordings from the apical 4-chamber view with the cursor placed at the free wall of the tricuspid valve [12]. Peak velocities of early (E) and late (A) diastolic filling and deceleration time were derived from transmitral Doppler profile [2]. Doppler tissue imaging was taken from medial mitral annulus and analyzed for early (E') and late (A') diastolic peak velocities [4]. Mitral E/E' ratio was subsequently calculated.

Inter- and intraobserver correlation for echocardiographic measurements variables reached in our laboratory 0.92 and 0.96, respectively. For assessment of pulmonary artery pressure (PA sys), the systolic pressure gradient between right atrium and ventricle was obtained from the tricuspid regurgitant jet envelope; and the value of 5 mmHg for right atrial pressure was added [13].

### Diagnosis of Diastolic Dysfunction

According to the diagnostic flowchart [3], diastolic dysfunction was assumed when the E/E' ratio exceeded 15 as derived from tissue Doppler. In case, tissue Doppler yielded an E/E' ratio ranging from 8 to 15, additional non-invasive parameters had to be fulfilled: left atrial volume index > 40 ml/m^2 ^body surface, or left ventricular mass index > 122 g/m^2 ^body surface, or transmitral E/A ratio < 0.5 plus deceleration time > 280 ms, or blood level of brain natriuretic peptide > 200 pg/mL.

### Statistics

Continuous variables were compared between groups using an unpaired *t *test (for normally distributed variables) or *Mann-Whitney U *test (for non-normally distributed variables). *Chi-square *analysis was used to compare categorical variables. All reported probability values were 2-tailed, and p < 0.05 was considered statistically significant. Stepwise logistic regression analysis was used to estimate the impact (odds ratio, 95% confidence interval) of the variables history of hypertension, systolic and diastolic blood pressure, heart rate, body mass index (BMI), BNP, the echocardiographic parameters E/E' ratio, left atrial volume index (LAVI), left ventricular endsystolic/enddiastolic volume indices (LVESVI/LVEDVI), left ventricular mass index (LVMI), right atrial diameter, systolic pulmonary pressure, and TAPSE on the dependent variable dyspnea (SPSS Inc., version 18).

## Results

The final study group with 291 participants was homogenous regarding sex (all female), age (range 69 to 79 years), heart rhythm (all in sinus rhythm), and left ventricular systolic function and size (LV EF > 50%, LVEDVI < 97 mL/m^2 ^body surface).

**Figure 1:**
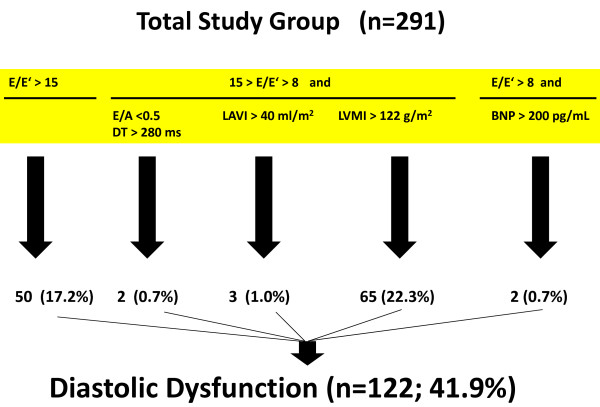
**Definitons for diastolic dysfunction**.

According to the diagnostic flowchart "How to diagnose HFNEF", parameters derived from tissue Doppler and laboratory findings (BNP levels) were concordant with the presence of diastolic dysfunction in 122 participants (41.9%). The diagnosis of diastolic dysfunction based in the majority of cases only on two criteria: in about 17% of cases (50/291) on the criterion "E/E' ratio > 15", and in about 22% of cases (65/291) on the criterion "15 > E/E'>8 and LVMI > 122 g/m^2^" (Figure 1).

The participants with diastolic dysfunction had on average a higher body mass index (28.34 ± 4.50 vs. 27.01 ± 4.55 kg/m^2^; p = 0.014), more frequent a history of arterial hypertension (82 vs. 63.9%; p = 0.001) and of hospitalization for congestive heart failure (13.1 vs. 5.3%; p = 0.033), poorer quality of life (9.77 ± 10.52 vs 6.84 ± 8.11 score; p = 0.008), and higher BNP blood levels (86.18 ± 178.92 vs. 51.79 ± 50.4 pg/mL; p = 0.018) as compared to those participants without signs of diastolic dysfunction. The number of participants complaining exertional dyspnea, however, was similar distributed among the subgroups with and without signs of diastolic dysfunction (40.2 vs 40.8%; p = n.s) (Table 1).

**Table 1:** Comparison of participants with and without diastolic dysfunction

	No Diastolic Dysfunction	Diastolic dysfunction	
**Parameter**	**n = 169**	**n = 122**	**p**

Age, years	74.07 ± 2.53	74.46 ± 2.72	0.210

Body mass index, kg/m^2^	27.01 ± 4.55	28.34 ± 4.50	0.014

Hypertension, n (%)	108 (63.9)	100 (82.0)	0.001

Diabetes mellitus, n (%)	15 (8.9)	12 (9.8)	0.941

Current smoking, n (%)	8 (4.7)	1 (1.2)	0.119

Dyslipidemia, n (%)	85 (50.3)	70 (57.4)	0.282

Myocardial infarction, n (%)	8 (4.7)	11 (9.0)	0.145

Hospitalization for CHF, n (%)	9 (5.3)	16 (13.1)	0.033

Stroke, n (%)	4 (2.4)	6 (4.9)	0.394

MLHFQ (score)	6.84 ± 8.11	9.77 ± 10.52	0.008

Dyspnea, n (%)	69 (40.8)	49 (40.2)	0.994

BNP, pg/mL	51.79 ± 50.4	86.18 ± 178.91	0.018

LVEF, %	68.6 ± 6.8	68.1 ± 6.0	0.516

Values are mean ± SD if not otherwise noted; CHF = congestive heart failure, MLHFQ = Minnnesota living with heart failure questionnaire, BNP = brain natriuretic peptide, LVEF = left ventricular ejection fraction

Participants with diastolic dysfunction complained about dyspnea in 49 cases (40.2%). It was hypothesized that the participants with dyspnea suffer from a more advanced stage of diastolic dysfunction, i.e. the parameters used to define diastolic dysfunction should be more abnormal as compared to cases without dyspnea. Table 2 compares the "severity" for diastolic dysfunction in both subgroups with (n = 49) and without dyspnea (n = 73). The "diagnostic key parameters" derived from Doppler echocardiography/tissue Doppler (E/A ratio, DT, E/E' ratio) revealed on average no significant differences; however the groups differ with respect to BNP, LAVI and LVMI.

**Table 2:** Presence of diastolic dysfunction - comparison of participants with and without dyspnea

	No Dyspnea	Dyspnea	
Parameter	n = 73	n = 49	p
BNP, pg/mL	57.1 ± 63.1	164.2 ± 266.6	0.023
LAVI, ml/kg^2^	21.7 ± 6.4	26.8 ± 9.1	0.001
LVMI, g/kg^2^	132.2 ± 26.0	141.7 ± 27.8	0.048
E/A ratio	0.85 ± 0.31	0.95 ± 0.44	0.139
DT, sec	245.7 ± 58.3	239.0 ± 65.7	0.556
E/E' ratio	14.2 ± 3.7	15.3 ± 6.1	0.237

Values are mean ± SD; LAVI = left atrial volume index; LVMI = left ventricular mass index; E = early diastolic filling peak velocity; A = late diastolic filling peak velocity; DT = deceleration time; E/E' ratio = ratio early diastolic filling velocity/mitral annular peak velocity

A logistic regression model was used to disclose the parameters which predict best dyspnea in participants with diastolic dysfunction. The parameters age, LV ejection fraction and LV volume indices were not added to this analysis, because these parameters were used as pre-selection criteria. The parameters history of hypertension, systolic and diastolic blood pressure, heart rate, the echocardiographic parameters E/E' ratio and TAPSE did not show a significant association with the variable dyspnea (data not shown). Dyspnea was predicted best by the systolic pulmonary artery pressure, followed by left atrial volume index, BNP, body mass index, and the parameters right atrial diameter and LV mass index (Table 3).

**Table 3:** Logistic regression analysis, dependent variable dyspnea

Parameter	Odds Ratio	P	95% Confidence Interval
PA sys	1.249	.000	1.113 - 1.400
LAVI	1.098	.001	1.037 - 1.162
BNP	1.011	.001	1.004 - 1.109
BMI	1.151	.002	1.053 - 1.257
Septal thickness	1.404	.009	1.089 - 1.811
RAD	1.126	.014	1.025 - 1.238
LVESDI	1.074	.026	1.009 - 1.145
LVMI	1.018	.027	1.002 - 1.033

PAsys = systolic pulmonary artery pressure; RAD = right atrial diameter; BMI = body mass index; LVESDI = left ventricular endsystolic diameter index; for other abbreviations see tables 1&2; for parameters LAVI, BMI, septal thickness, RAD, LVESDI and LVMI were measurements available in 122 participants; measurements were available for PAsys and BNP in 50 and 99 participants, respectively

## Discussion

Diastolic heart failure is characterized by impaired left ventricular relaxation, increased left ventricular stiffness, increased interstitial deposition of collagen, and modified extracellular matrix proteins [1,3]. It has been estimated that approximately 50% of the heart failure population has a normal left ventricular ejection fraction, and diastolic heart failure may be - at least partially - responsible for heart failure signs and symptoms [1]. Compared with patients with heart failure and reduced LV ejection fraction, individuals with HFNEF are typically older, more likely women, and have a higher likelihood of arterial hypertension [14]. The prognosis of patients with HFNEF appears to be only hardly better than that of individuals with heart failure and impaired LV systolic function [15,16]. While life expectancy increasing, HFNEF may be a growing health problem.

A prerequisite in the diagnosis of HFNEF is the evidence of diastolic dysfunction. The reported prevalence of LV diastolic dysfunction in the general population varies from 11.1 to 34.7% [6,17-20], and may depend on various factors, in particular the characteristics of the population studied, and the criteria applied to define diastolic dysfunction. In the present cohort of preselected elderly (age range 69-79 years) women with a normal LV ejection fraction and a normal LV geometry, the rate of diastolic dysfunction was as high as 42%. In agreement to previous studies [6,19,20] diastolic dysfunction was associated with a higher body mass index, more frequent with a history of arterial hypertension, a poorer quality of life and higher BNP blood levels as compared to those participants without signs of diastolic dysfunction.

A key element in the diagnosis of HFNEF is the evidence of elevated LV filling. Three ways have been proposed to diagnose raised LV filling pressures: a. invasive measurements, b. unequivocal tissue Doppler measurements, and c. a combination of Doppler echocardiographic parameters and elevated BNP blood levels [3]. Conventional Doppler echocardiographic indices (E/A ratio derived from mitral inflow and pulmonary venous flow patterns) have clear limitations and rarely allow the accurate differentiation between normal from pseudonormal mitral inflow pattern [1]. In the present study we used for diagnosing diastolic dysfunction the flowchart as proposed by the Heart Failure and Echocardiography Associations of the ESC [3]. We focused on the non-invasive arm of the flowchart because of practicability in a population-based survey. This non-invasive arm of the flowchart emphasizes the E/E' ratio derived from tissue Doppler as one key element of diagnosis of diastolic dysfunction with an E/E' ratio > 15 being unequivocal for elevated LV filling pressures. In the present study population, about 17% of cases alone fulfilled this criterion (41% of total subgroup with evidence of diastolic dysfunction). An E/E' ratio between 8 and 15 appears suggestive but nondiagnostic for diastolic dysfunction, and the flowchart demands further measurements. In the present study we used some but not all criteria proposed by the flowchart: we ignored the criterion "presence of atrial fibrillation", because sinus rhythm was an inclusion criterion, as well as the criterion "difference between duration of reverse pulmonary vein atrial systole flow and duration of mitral valve atrial flow > 30 ms", because the pulmonary vein flow pattern was not routinely assessed in our study population. The combined criterion "15>E/E'>8 and left ventricular mass index > 122 g/m^2 ^(for women)" was positive in our study cohort in 22.3% of cases (53% of total subgroup with evidence of diastolic dysfunction). Taken together, the diagnosis of diastolic dysfunction based in our study population in 94% of cases on two criteria only.

The flowchart allows an alternative pathway to diagnose diastolic dysfunction: the use of the biomarkers brain natriuretic peptide (BNP) or NT-proBNP in first line, in combination with an E/E' ratio > 8. In our study population, 7.6% of participants fulfilled this criterion (data not shown). This number is in sharp contrast to the above mentioned cases with diastolic dysfunction diagnosed by tissue Doppler indices in first line (42%).

Somewhat unexpected, the diagnosis of diastolic dysfunction (using tissue Doppler in first line for diagnosis) showed only a poor association with the symptom "dyspnea". In fact, approximately 40% of elderly women in the present study cohort complained dyspnea: this symptom, however, was similar distributed among the subgroups with and without signs of diastolic dysfunction. The "key parameters" for the diagnosis of diastolic dysfunction (E/A ratio and deceleration time; E/E' ratio) did not further discriminate those women with diastolic dysfunction and dyspnea. Additional echocardiographic parameters such as the systolic pulmonary pressure, left atrial volume index, right atrial diameter, and left ventricular mass index were more helpful to characterize participants with dyspnea. This observation suggests that the demonstration of elevated LV filling pattern by tissue Doppler alone may be not sufficient to "explain" the symptom dyspnea in elderly patients, and other factors have to be taken into consideration. Several causes of dyspnea in elderly have been described [21]. Shortness of breath may be associated with older age, poor perceived health, anxiety and depressive symptoms, impaired daily functioning, lower happiness or muscular disabilities. Our data does not allow discriminating between two possibilities: a. the additional factors may be related to a more advanced stage of diastolic dysfunction with secondary structural and hemodynamic changes, b. the additional factors are the result of co-morbidities such as left ventricular hypertrophy or pulmonary arterial hypertension. Since the magnitude of the E/E' ratio did not correlate with the symptom dyspnea in women classified for diastolic dysfunction, our data favors the latter suggestion.

As a limiting factor we have to consider, that we can not thoroughly generalize our data to the community because of the study exclusion criteria. However, we wanted to analyze the interplay between diastolic function and dyspnea without apparent underlying diseases.

## Conclusion

In summary, our population-based cross-sectional study in elder women confirms a high percentage of participants with signs of diastolic dysfunction, as assessed by the actual diagnostic guidelines of the ESC. According to the guidelines, the diagnosis of diastolic dysfunction based in the majority of cases on two criteria (E/E' ratio > 15, 15>E/E'>8 and LVMI > 122 g/m^2^). The demonstration of diastolic dysfunction showed only a poor association with the symptom dyspnea. Additional structural or hemodynamic changes are necessary to "explain" the symptom dyspnea in this cohort of elderly women. It is unclear whether these additional factors are secondary to a more advanced stage of diastolic dysfunction, or are related to cardiovascular co-morbidities, or both.

## Abbreviations

BMI: body mass index; BNP: plasma B-type (brain) natriuretic peptide; CCS: Canadian Chest Society; DT: deceleration time; EF: ejection fraction; HFNEF: heart failure with normal ejection fraction; LAVI: left atrial volume index; LV: left ventricular; LVESVI: left ventricular endsystolic volume index; LVEDVI: left ventricular enddiastolic volume index; LVMI: left ventricular mass index; MLHFQ: Minnesota living with heart failure questionnaire; PAsys: Systolic pulmonary artery pressure; PCI: percutaneous coronary intervention; RAD: right atrial diameter; TAPSE: tricuspid annular plane systolic excursion.

## Competing interests

The authors declare that they have no competing interests.

## Authors' contributions

AG, MG and AM had full access to all of the data in the study and take responsibility for the integrity of data and the accuracy of data analysis. Study concept and design: AG, AM. Acquisition of data: AG, MG. Analysis and interpretation of data: AG, MG, TS, AV, UR, UK, AM. Preparation of the manuscript: AG, MG, TS, AV, UR, UK, AM. All authors read and approved the final manuscript.

## Pre-publication history

The pre-publication history for this paper can be accessed here:

http://www.biomedcentral.com/1471-2318/11/71/prepub
